# Effect of DRD4 receptor −616 C/G polymorphism on brain structure and functional connectivity density in pediatric primary nocturnal enuresis patients

**DOI:** 10.1038/s41598-017-01403-1

**Published:** 2017-04-27

**Authors:** Bing Yu, Na Chang, Yao Lu, Hongwei Ma, Na Liu, Qiyong Guo

**Affiliations:** 1 Department of Radiology, Shengjing Hospital of China Medical University, Shenyang, 110004 China; 2 Center of Medical Laboratory Technology, China Medical University, Shenyang, 110001 China; 3 Department of Developmental Pediatrics, Shengjing Hospital of China Medical University, Shenyang, 110004 China; 4 Department of Radiology, The People’s Hospital of Liaoning Province, Shenyang, 110016 China

## Abstract

The dopamine D4 receptor (DRD4) promoter (−616; rs747302) has been associated with primary nocturnal enuresis (PNE); however, its relationship with neuroimaging has not been investigated. Therefore, we assessed the effects of the DRD4 −616 C/G single nucleotide polymorphism on the gray matter volume (GMV) and functional connectivity density (FCD) during resting-state functional magnetic resonance imaging in children with PNE using voxel-based morphometry and FCD methods. Genomic and imaging data were obtained from 97 children with PNE and 105 healthy controls. DRD4 −616 C/G was genotyped. Arousal from sleep (AS) was assessed on a scale of 1–8. Both the main effect of genotype and the group (PNE/control)-by-genotype interaction on GMV and FCD were calculated. Our results showed that C-allele carriers were associated with a higher AS, decreased GMV and FCD in the pregenual anterior cingulate cortex; children with PNE carrying the C allele exhibit decreased GMV and FCD in the thalamus; however, controls carrying the C allele exhibit increased FCD in the posterior cingulate cortex. These effects of genetic variation of the DRD4 locus may help us understand the genetic susceptibility of the DRD4 −616 C allele to PNE.

## Introduction

Although often neglected by child psychiatry until now, primary nocturnal enuresis (PNE) is common in childhood, affecting up to 20% of children and nearly 2% of young adults^1^. It is defined as continuous, involuntary and nocturnal enuresis associated with normal daytime urination beyond the age of five. PNE may cause significant psychosocial stress and impairment of self-esteem in pediatric patients and frustrate their caregivers.

Genetic factors play an important role in PNE. The molecular genetic approach has been employed to understand the causes of enuresis since 1995, and markers have been found on chromosomes 12q, 13q13–q14.3 (ENUR1) and 22q11 (ENUR3)^2, 3^. Our previous study suggested that the frequency of the C allele in the dopamine D4 receptor (DRD4) promoter (−616; rs747302) was significantly higher in PNE patients^4^. This single nucleotide polymorphism (SNP) in the DRD4 promoter may lead to a reduction of DRD4 protein in the thalamus, limbic system, frontal cortex, and globus pallidus^5^. However, to the best of our knowledge, no candidate genes for PNE susceptibility in these regions have been identified until now.

In the past decades, a few functional neuroimaging studies have been performed on PNE patients. These studies suggested that there are functional and structural differences in multiple brain regions in the patients compared to healthy children^1, 6–10^. Our initial whole brain functional connectivity study suggested that cerebello-thalamo-frontal circuit abnormalities are likely involved in the onset and progression of attention impairment in pediatric PNE patients^1^. However, the relationship between genetic polymorphisms and neuroimaging abnormalities has not been combined.

Therefore, the purpose of the study was to assess effects of the DRD4 −616 C/G SNP on the gray matter volume (GMV) and resting state functional connectivity (rsFC) in children with PNE and healthy controls using voxel-based morphometry (VBM) and functional connectivity density (FCD) methods. Both the effect of genotype and the group-genotype interaction were evaluated in the VBM and FCD analyses.

## Results

### Demographic, genetic, and behavioral characteristics

Four children from the PNE group and two from the control group were also excluded for failing to complete MRI scans. The complete genetic and fMRI data of the remaining 97 children in the PNE group (M:F = 57:40; average age 10.4 ± 1.5 y; 4.8 ± 0.8 y of education) and 105 children in the control group (M:F = 58:47; average age 10.3 ± 1.2 y; 4.7 ± 0.8 y of education) were included in the study. There were no significant differences between genotype (C/G) with regard to gender, age, years of education, full-scale intelligence quotient (FIQ) scores or framewise displacement (FD) (Tables 1 and 2).

**Table 1:** Demographic and behavioral data^a^.

Characteristic	PNE (*n* = 97)		Control (*n* = 105)	
	C	G	C	G
Gender (M:F)	29:20	28:20	28:22	30:25
Age (y)	10.3 ± 1.6	10.5 ± 1.4	10.5 ± 1.0	10.1 ± 1.4
Years of education	4.9 ± 0.5	4.7 ± 0.9	4.8 ± 1.0	4.6 ± 0.5
FIQ	104 ± 11.0	106 ± 10.9	104 ± 9.9	106 ± 13.4

Abbreviations: PNE, primary nocturnal enuresis; FIQ, Full-scale Intelligence Quotient.

^a^Data are presented as the mean ± standard deviation.

^b^No significant differences are found in any measures between GG and GC/CC individuals.

**Table 2:** Assessment of head motion using framewise displacement (FD).

Group	Control		PNE		F(P)*		
Genotype	C	G	C	G	Main effect of group	Main effect of genotype	Genotype ×group
n	50	55	49	48	(1,198)		
FD	0.096	0.087	0.092	0.088	0.0304	0.5710	0.0845
	(0.054)	(0.050)	(0.076)	(0.062)	(0.8617)	(0.4508)	(0.7716)

Abbreviations: PNE, primary nocturnal enuresis.

The data are shown as the means (standard deviation).

*F(P) is based on the analysis of variance with genotype and group as two factors.

**Figure 1:**
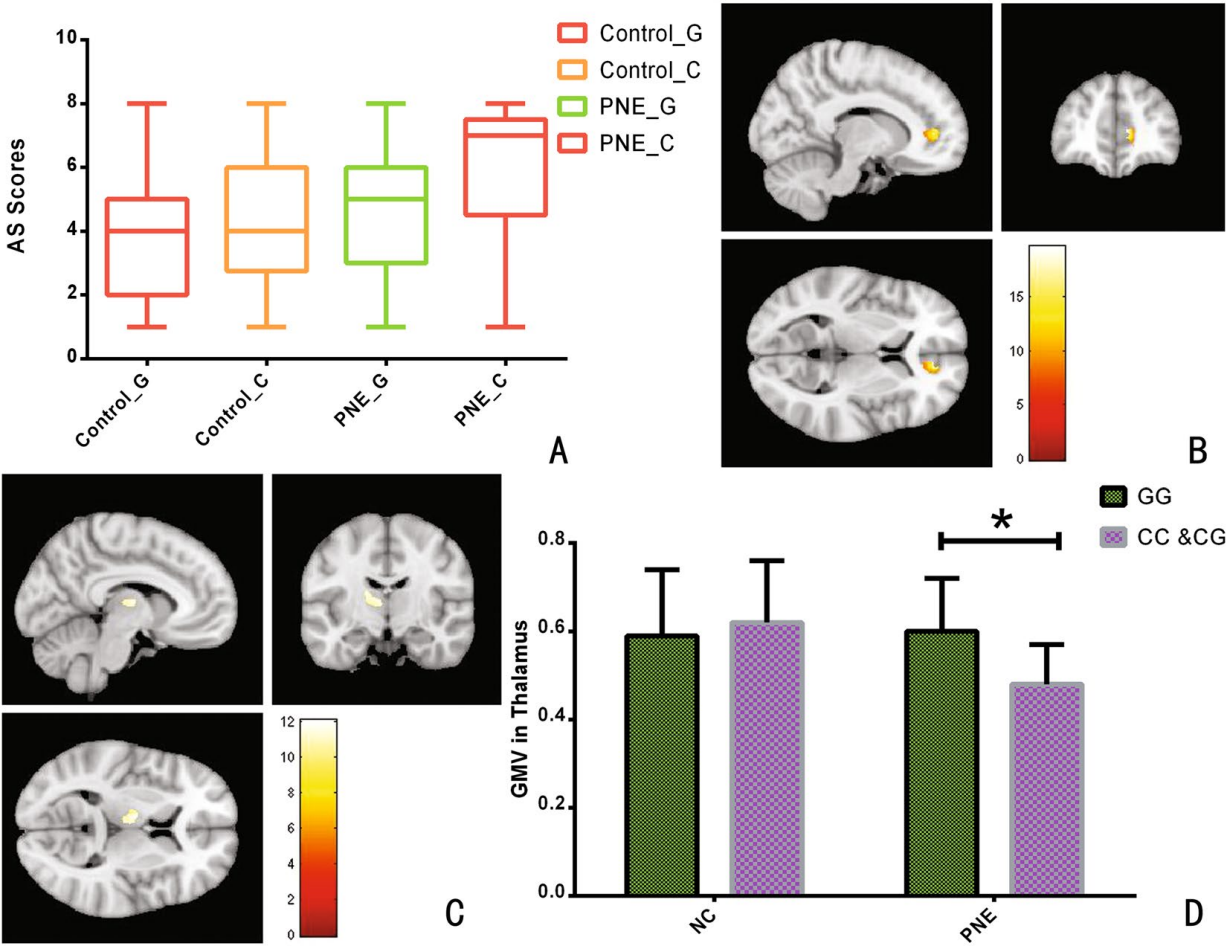
Association between DRD4 −616 C/G and AS and GMV. (**A**) Comparisons of AS scores in different genotypes and groups. (**B**) Whole-brain thalamic VBM analysis demonstrates a significant main effect of genotype in the pregenual ACC. (**C**) Whole-brain thalamic VBM analysis demonstrates a significant interaction between genotype and group in the thalamus. (**D**) PNE C-allele carriers exhibited a significantly decreased GMV in the pregenual ACC compared to the PNE G-allele homozygotes.

Arousal from sleep (AS) scores of the PNE group were significantly higher than those of the healthy control group (Z = 4.930, ties corrected, *P* = 0.0015). When AS scores were compared within the PNE group by genotype, the AS scores of C-allele carriers were significantly higher than GG homozygotes (Z = 2.543, ties corrected, *P* = 0.0110); however, there were no significant differences in AS scores between the genotypes in the control group (Z = 1.564, ties corrected, *P* = 0.1179) (Fig. 1A).

### Differences in GMV based on genotype

A main effect of genotype on GMV in the pregenual anterior cingulate cortex (ACC) (peak Montreal Neurological Institute [MNI] coordinates: x = 12, y = 41, z = 6; cluster size = 267 voxels; peak F_1,198_ = 17.15, *P* < 0.05, false discovery rate [FDR] corrected) (Fig. 1B) and a group-genotype interaction in the thalamus (peak MNI coordinates: x = −8, y = −11, z = 7; cluster size = 295 voxels; peak F_1,198_ = 12.36, *P* < 0.05, FDR corrected) were found (Fig. 1C).

We then extracted the GMV from the cluster that exhibited interaction and compared them between the two genotypes within the PNE and control groups. Post-hoc testing demonstrated that the C-allele carriers in the PNE group had significantly decreased GMV in the thalamus (*t* = −5.538, *P*_adjusted_ < 0.001) compared with the GG homozygote patients (Fig. 1D).

### Differences in FCD based on genotype

**Figure 2:**
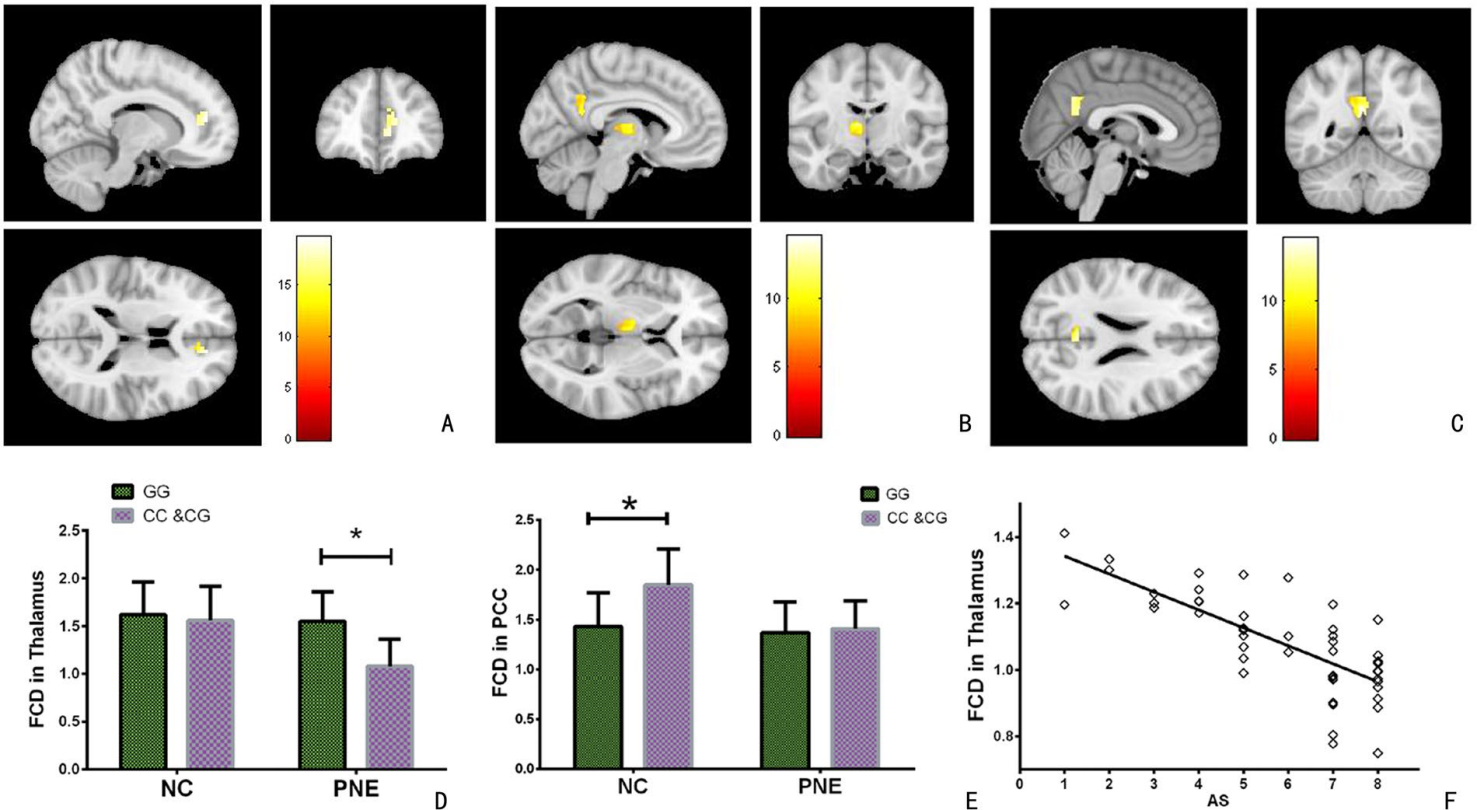
Association between DRD4 −616 C/G, AS and FCD. (**A**) A main effect of genotype on the FCD was found in the pregenual ACC. (**B**) FCD analysis demonstrates a group-genotype interaction in the thalamus. (**C**) FCD analysis demonstrates a group-genotype interaction in the PCC. (**D**) Post-hoc testing demonstrated that the C-allele carriers in the PNE group had a significantly decreased FCD in the thalamus. (**E**) Post-hoc testing revealed that controls carrying the C allele exhibited increased FCD in the PCC. (**F**) The FCD of the thalamus also showed a significant negative correlation with the AS scores in C-allele carriers of the PNE group.

A main effect of genotype on the FCD was found in the pregenual ACC (peak MNI coordinates: x = 10, y = 42, z = 7; cluster size = 227 voxels; peak F_1,198_ = 17.93, *P* < 0.05, FDR corrected). In addition, group-genotype interaction in the thalamus (peak MNI coordinates: x = −8, y = −10, z = 10; cluster size = 249 voxels; peak F_1,198_ = 14.53, *P* < 0.05, FDR corrected) and posterior cingulate cortex (PCC) (peak MNI coordinates: x = −3, y = −51, z = ; cluster size = 87 voxels; peak F_1,198_ = 13.55, *P* < 0.05, FDR corrected) were found (Fig. 2A–C).

We then extracted the FCD from the thalamus and PCC, and compared it between the two genotypes within the PNE and control groups. Post-hoc testing demonstrated that the C-allele carriers in the PNE group had a significantly decreased FCD (*t* = −5.183, *P*_adjusted_ < 0.01) compared with the GG homozygote patients in the thalamus. However, controls carrying the C allele exhibited increased FCD in the PCC (*t* = 4.336, *P*_adjusted_ = 0.01). The FCD of the thalamus also showed a significant negative correlation with the AS scores in C-allele carriers of the PNE group (*r* = −0.696, *P* < 0.001) (Fig. 2D–F).

## Discussion

A difficulty with AS is a prerequisite for PNE, and has been proven in PNE patients through questionnaires, auditory signals and electroencephalographic analysis^11^. This study also demonstrated that the AS scores of C-allele carriers were significantly higher than GG homozygotes in the PNE group.

Our results demonstrated that C-allele carriers exhibit decreased GMV and FCD in the ACC. We speculate that the C allele may disrupt the activator protein 2 (AP-2) binding site in the promoter region of the *DRD4* gene. Since AP-2 is a sequence-specific mammalian transcription factor that can activate gene transcriptio^12^, the mutation may lead to down regulation of DRD4 transcription. Consequently, the decrease of dopamine D4 receptors may affect the modulating synaptic plasticity and result in functional and structural abnormalities and cause the abnormalities in the ACC^13^.

Previous tract-tracing studies have identified the pregenual ACC as the central node of the cortical and subcortical systems within the medial prefrontal cortex (mPFC), and the majority of the sleep ‘active’ and sleep ‘inactive’ neurons are located in the pregenual ACC, suggesting that the pregenual ACC is the central hub in the functional architecture of the mPFC with regard to the sleep cycle. Therefore, the morphometry and functional defects in the pregenual ACC may inhibit children carrying the DRD4 −616 C allele to wake from sleep, making then more susceptible to nocturnal enuresis.

Disorder in the GMV and FCD within the thalamus were shown in C-allele carrying PNE children in the present study. We speculate that the C allele-related decrease in the DRD4 receptor may weaken the inhibition of depolarization evoked by Ca^2+^-dependent γ-aminobutyric acid (GABA) release, causing increased GABA concentrations in the thalamus^14, 15^. This abnormal accumulation of GABA may affect the structure and function of the thalamus.

The thalamus plays an important role in the switch between sleep and waking, relaying sensory afferent signals from the bladder and transmitting them through the periaqueductal gray, to the ACC, the insula, and the lateral prefrontal cortex^16^. Some previous imaging studies have also shown that the strength of thalamo-cortical connectivity was closely related to vigilance and external conscious perception^17, 18^. Thus, the altered GMV and FCD in the thalamus may result in an inability to wake during sleep in response to the need to void, and this is consistent with abnormally large urine volume in the bladders of children with PNE^19^.

However, regarding these genotypic differences in the thalamus in PNE subjects, this group-dependent mechanism may be because the structure and function of the thalamus were affected by multiple neurotransmitters and because of the complex afferent connections of the thalamus. Therefore, the presumed changes in GABA concentrations may be compensated by other neurotransmitters in C-allele control children and only result in obvious abnormalities in PNE patients.

C allele-carrying controls in our study were found to have increased FCD in the PCC. This may be a compensatory mechanism within the default mode network (DMN). The DMN is indispensable for integrating whole-brain regions in a fully conscious awake state. An increasing number of studies have suggested that the DMN plays a key role in generating conscious awareness^20, 21^. Thus, it is essential for consciousness, both for integrating brain activity and for efficient communication between regions in the DMN and the rest of the brain^22^.

Since the pregenual ACC is the key node of the anterior DMN (aDMN), the structural and FCD abnormalities may affect the function of the aDMN, and consequently affect the functional connectivity to other brain areas. However, as the key node of the posterior DMN (pDMN), the increase of FCD in the PCC may relieve the communication disorders due to functional disorders related to the pregenual ACC and maintain the normal arousal threshold in C allele-carrying controls.

The GMV and FCD abnormalities described here provide the initial evidence to support these variations in children with PNE; however, this study had some limitations that necessitate further research to validate these findings and the proposed mechanism. The sample size and subject variety were insufficient to concretely prove that this hypothesis is correct, and require future testing on larger, more varied cohorts. Furthermore, we combined subjects with CC and CG genotypes as a group of C-carriers rather than combining subjects with GG and GC genotypes into a group of G-carriers. Thus, the difference in GMV and FCD between PNE patients with CC and CG genotypes needs to be further investigated. Third, we cannot completely simulate the circumstance and process of natural nocturnal enuresis and record the fMRI signal changes simultaneously, so the relationship between sleep disorder and abnormalities found in our study needs more evidence. Fourth, the resting state cannot be considered a static condition. It is associated with spontaneous thoughts and random uncontrolled cognitive processing, which must affect functional measures to some degree. Finally, in an effort to avoid possible confounding effects of abnormal brain structure and function caused by attention-deficit/hyperactivity disorder (ADHD) on experimental results, subjects exhibiting PNE comorbid with ADHD were excluded from this study. Such exclusion may incur certain selection bias that must be considered in reviewing these results and in the design of future studies.

In summary, we found decreased GMV and FCD in the pregenual ACC; decreased GMV and FCD in the thalamus in C allele-carrying PNE children and increased FCD in the PCC in C allele-carrying controls. These effects of genetic variation of the DRD4 locus may help us understand the genetic susceptibility of the DRD4 −616 C allele to PNE.

## Methods

### Participants

One hundred and four pediatric PNE patients (9.1–11.9 years, with a median age of 10.4 years) and 107 healthy controls (9.0–11.8 years, with a median age of 10.1 years) were enrolled in this study. The present sample was pre-stratified according to DRD4 −616 C/G SNP (see below for genotyping and Table 1 for demographic and psychometric characteristics).

The protocol used for this study was approved by the Ethics Committee of Shengjing Hospital of China Medical University, and all the parents or legal guardians of all participants provided written informed consent according to institutional guidelines. The study methods were conducted in accordance with the approved guidelines.

All children in the PNE group met the inclusion criteria according to the International Children’s Continence Society: urination under control during the daytime, involuntary urination during sleep at least twice a week for more than 6 months, normal blood and urine biochemistry, and normal urine culture and flowmetry. In addition, ultrasound examination of the urinary tract revealed no abnormal kidney or urinary tract defects, residual urine, or other urological or neurological disorders or abnormalities^23^. All included subjects underwent routine MRI examination and had normal results.

Prior to inclusion, all children regularly attended the enuresis outpatient clinic, and no children were included who had previously been treated with any typical or atypical psychoactive drug.

Each subject completed the Childhood Behavior Checklist as a screening tool and then underwent a structured diagnostic interview by a child psychiatrist. Children with a current or historic diagnosis of any neurological or psychiatric disease according to the Diagnostic and Statistical Manual of Mental Disorders published by the American Psychiatric Association (DSM-IV), especially ADHD, were excluded from the study. The FIQ was also measured for each study participant. A questionnaire was used to AS on a scale of 1–8 according to the methodology of Chandra *et al*.^24^ (see Supplementary Information for details of the AS SCORING SYSTEM).

### Genotyping

Standard protocols were used to extract DNA directly from blood lymphocytes using the EZgene TM Blood gDNA Miniprep Kit (Biomiga Inc., San Diego, CA, USA). The DRD4 −616 C/G SNP was genotyped by polymerase chain reaction-restriction fragment length polymorphism (PCR-RFLP)^25^ on an ABI 9700 Gene Amp PCR System (Perkin-Elmer, Norwalk, CT, USA). The PCR analysis used a native Pfu polymerase (Stratagene, La Jolla, CA, USA), and the forward primer (5′-TCTTCTGCACGTTTGGAACCTACCC-3′) and the reverse primer (5′-CTCAACCGCCGACGCCTAGCTCACTC-3′). Amplification consisted of incubation at 98 °C for 1 min followed by 35 cycles at 98 °C for 20 s, 68 °C for 30 s, and 74 °C for 2 min. Quality control of the data was performed by duplicating one subset of DNA samples randomly; the reproducibility was 100%. Subjects that we failed to genotype were excluded from further experiments. Given the low frequency of subjects who were homozygous for the C allele of the −616 SNP, participants were classified as having zero, one, or two copies of the G allele for statistical analyses.

### Statistical analyses of demographic and genetic data

SPSS 17.0 software (SPSS Inc., Chicago, IL, USA) was used to analyze the demographic and genetic data. Assumptions for normality were tested for continuous variables (age, educational years and FIQ) using the Kolmogorov–Smirnov test. All variables were normally distributed. An analysis of variance (ANOVA) with genotype and group as fixed factors was used to examine differences in continuous variables (age, educational years and FIQ). All statistical analyses had a two-tailed α level of less than 0.05 for statistical significance.

We performed three sets of AS score comparisons using the Mann–Whitney U test method: (1) between all PNE patients and controls; (2) between the C-allele carriers and GG homozygotes in the PNE group; (3) between C-allele carriers and GG homozygotes in the control group. A P-value of less than 0.016 was considered statistically significant after using Bonferroni corrections for multiple comparisons.

### Imaging data acquisition

All imaging studies were performed using a 3.0 Tesla MR scanner (Intera Achieva; Philips Medical Systems, Best, Netherlands) with an eight-channel Sensitivity Encoding (SENSE) head coil. High-resolution 3D Turbo Field Echoing T1WI structural image scanning was applied. The following parameters were used: TR/TE: 9.6 ms/4.6 ms; FOV: 230 mm; matrix: 256 × 256; layers: 128; and section thickness: 1.2 mm. fMRI data were acquired with a three-dimensional principle of echo shifting with a train of observations (3D-PRESTO) pulse sequence (TR/TE: 16/28 ms; FOV: 230 mm; matrix: 64 × 64; layers: 29; slice thickness: 4 mm; acquisition time per volume was 1 s). The scanning plane was parallel to the anterior/posterior commissure (AC-PC) line. The duration of the dummy scan was 8 s and was added at the start of each fMRI scan in order to eliminate artifacts induced by chemical shifts and to stabilize the magnetic field. Thus, each fMRI scan was performed over 488 s. While scanning, a thick ear cushion was added to fix the placement of the head, to minimize the effects of constructed defects in the test data caused by head movements.

### Structural data post-processing

The high-resolution T1-weighted structural images were subjected to VBM analysis. This was completed using the VBM8 tools (http://dbm.neuro.uni-jena.de/vbm/). Preprocessing of VBM data was performed using the SPM8 software package (Wellcome Trust Centre for Neuroimaging at UCL, London, UK), a package based on the MATLAB (MathWorks, Natick, MA, USA) platform. Initially, T1-weighted structural images were Diffeomorphic Anatomical Registration Through Exponentiated Lie (DARTEL) algebra-normalized to the standard stereotaxic space of the MNI (http://www.mni.mcgill.ca/) including modulation to preserve regional signal intensities, and re-sliced to 1.5 mm × 1.5 mm × 1.5 mm. The normalized data were then segmented into gray matter, white matter and cerebrospinal fluid. Subsequently, images were smoothed with a 6 mm full-width at half maximum Gaussian kernel.

### Statistical analyses of structural data

To determine the effects of group and genotype on GMV, a two-way analysis of covariance (ANCOVA) on a voxel-by-voxel basis was performed with groups (PNE patients and healthy controls) and genotypes (non-GG or GG) on GMV, age, educational years and FIQ were included in the model as additional covariates. The FDR approach was applied to identify the restriction threshold capable of reducing the proportion of type I errors to <0.05. The voxel-wise statistical threshold was at a P-value of <0.05 (FDR corrected) and a minimal cluster size of 50 voxels. We were interested in both the main effect of genotype and the group-by-genotype interaction. If the interaction was significant, post-hoc comparison of the GMV in the clusters showed interaction were performed to determine the genotypic differences in the PNE and control groups respectively. Correction for multiple comparisons was performed using Bonferroni correction methods.

### fMRI data preprocessing

All fMRI data preprocessing was performed using the SPM8 software package (Wellcome Trust Centre for Neuroimaging at UCL) on the MATLAB (MathWorks) platform. The data were first corrected for acquisition time delays between slices. Head motion parameters were estimated and the corresponding corrections were performed using a six-parameter rigid-body transformation. Participants were excluded from further analysis if their maximum displacement in any direction (x, y, z) was more than 2 mm, or their maximum rotation (x, y, z) was greater than 2.0°. Framewise displacement (FD) was also calculated to assess the changes in head position obtained from derivatives of the rigid body realignment estimates that are used to realign fMRI data^26, 27^. Images were then resampled to 3 mm × 3 mm × 3 mm voxels in MNI-labeled space (Bounding Box: [−90 −126 −72; 90 90 108]) and smoothed with an isotropic Gaussian kernel (full width at half maximum: 8 mm). The resultant data were further filtered using a bandpass temporal filter (0.01–0.1 Hz) to reduce the effects of low frequency drift and high frequency physiological noise using the Resting-State fMRI Data Analysis Toolkit (REST) software package (http://restfmri.net/forum/REST_V1.8); nuisance signals such as head motion parameters and average blood-oxygen-level dependent (BOLD) signals of the ventricular and white matter were regressed out. ANOVA with genotype and group as fixed factors was used to examine differences in FD^28^.

### FCD analysis

The FCD value of each voxel was calculated using the REST software package according to the method described by Tomasi *et al*.^29^. Pearson’s linear correlation was used to calculate the functional connections between voxels restricted within regions of gray matter. The two voxels with a correlation coefficient of more than 0.5 were considered functionally connected. The global FCD of a given voxel was computed as the number of functional connections between it and all other voxels within the GM regions. The FCD value of each voxel was then divided by the mean FCD value of the whole brain (grand mean scaling) for each participant in order to increase the normality of the FCD distribution. The resultant images were spatially smoothed with a 6 mm × 6 mm × 6 mm Gaussian kernel.

A two-way ANCOVA on a voxel-by-voxel analysis was also performed with groups (PNE patients and healthy controls) and genotypes (non-GG or GG) on FCD. Age, educational years, and FIQ were included in the model as additional covariates. Data from the brain regions demonstrating the SNP main effects and gene-grouping interactions were reported. The FDR approach was applied to identify the restriction threshold capable of reducing the proportion of type I errors to <0.05. The voxel-wise statistical threshold was also at a *P*-value of <0.05 (FDR corrected) and a minimal cluster size of 50 voxels.

To further explore the details within the clusters that showed significant effects of interactions, post-hoc comparisons of the FCD were performed to determine the genotypic differences in the PNE and control groups. Correction for multiple comparisons was performed using Bonferroni correction methods.

## Electronic supplementary material

Supplementary Information
