# Supplementary material for: Effect of DRD4 receptor −616 C/G polymorphism on brain structure and functional connectivity density in pediatric primary nocturnal enuresis patients
Source: Sci Rep. 2017 Apr 27;7:1226. doi: 10.1038/s41598-017-01403-1 (PMC5430843; doi:10.1038/s41598-017-01403-1)
Supplement: Supplementary file 1 — Supplementary Information [file 41598_2017_1403_MOESM1_ESM.doc]

**Supplementary Information**

**Effect of DRD4 receptor −616 C/G polymorphism on brain structure and functional connectivity density in pediatric primary nocturnal enuresis patients**

Bing Yu, Na Chang, Yao Lu, Hongwei Ma, Na Liu, Qiyong Guo

Bing Yu, Shengjing Hospital of China Medical University

**SCORING SYSTEM FOR ASSESSING AROUSAL FROM SLEEP**

Please choose the number which best describes your child’s arousal from sleep

1. Wakes up from the slightest noise or from turning on the light in the room

2. Wakes up when called by name gently

3. Wakes up when called by name loudly or sound of a bedside alarm clock

4. Wakes upon shouting the name at the ear or upon gentle shaking

5. Wakes up with loud noise and vigorous shaking

6. Wakes up when physically stood up

7. Wakes up when walked from the bed with support

8. Doesn’t awaken, has to be carried out of bed
